# A Randomised Controlled Trial Comparing the Effects of Personalised Diet and Physical Activity Intervention Versus Usual Care on Cardiometabolic Risk Factors in Adults with Inactive Inflammatory Bowel Disease

**DOI:** 10.3390/nu18050785

**Published:** 2026-02-27

**Authors:** Jia Min Yap, Catherine L. Wall, Kim Meredith-Jones, Ella Iosua, Hamish Osborne, Michael Schultz

**Affiliations:** 1 Department of Medicine, University of Otago, Dunedin 9011, New Zealand; kim.meredith-jones@otago.ac.nz (K.M.-J.); hamish.osborne@otago.ac.nz (H.O.); michael.schultz@otago.ac.nz (M.S.); 2 Health NZ Southern, Dunedin Hospital, Dunedin 9011, New Zealand; 3 Department of Medicine, University of Otago, Christchurch 8011, New Zealand; catherine.wall@otago.ac.nz; 4 Biostatistics Centre, University of Otago, Dunedin 9011, New Zealand; ella.iosua@otago.ac.nz

**Keywords:** inflammatory bowel disease, lifestyle intervention, diet, physical activity, cardiometabolic disease risk

## Abstract

**Background**: Adults with inflammatory bowel disease (IBD) have a high prevalence of modifiable cardiometabolic risk factors. This study investigates the impact of a personalised diet and physical activity intervention versus usual care on the risk factors. **Methods**: A 6-month randomised controlled trial was conducted at three hospitals in New Zealand (NZ) from 2023 to 2024. Adults with IBD in remission, a body mass index > 25 kg/m^2^, and a low fibre intake < 25 g/day were recruited. Participants were randomised to receive either generic healthy eating and physical activity education or personalised heart-healthy eating education based on the NZ Heart Foundation and a self-led physical activity program. The primary outcome was change in body fat, and secondary outcomes included disease activity, biomarkers, quality of life, physical activity, and dietary intake. Between-group differences were analysed using multivariable regression. **Results**: Sixty-four participants were randomised, and 51 (80%) completed the intervention. The median age was 47 years (LQ, UQ: 37, 55), 59% participants were female, 61% had Crohn’s disease, and 85% had faecal calprotectin < 150 µg/g. Common cardiometabolic risks were high waist circumference (88%) and abnormal lipid profile (56%). There were no significant differences in primary or secondary outcomes except for dietary intakes: increased fruit (0.5 serves/day; 95% CI: 0.1, 1.0) and dietary fibre (3.1 g/1000 kcal/day; 95% CI: 1.1, 5.1); reduced discretionary food and drink (−1.7 serves/day; 95% CI: −3.0, −0.3), and sodium (−911 mg/day; 95% CI: −1783, −40). **Conclusions**: Personalised dietitian advice led to meaningful dietary changes without exacerbating disease activity. More intensive activity modalities can be recommended to support body composition improvements.

## 1. Introduction

Diet and physical activity are important in managing IBD, positively influencing disease outcomes [1] and quality of life [2,3]. However, these lifestyle factors are also critical for managing cardiometabolic risk factors [4]. Emerging evidence shows that people with IBD have a higher prevalence of traditional cardiometabolic risk factors such as hypertension, central obesity, and hypertriglyceridemia compared to age-sex matched controls [5]. This may be related to a myriad of factors including chronic systemic inflammation, medications, and lifestyle challenges [6]. In adults with obesity and type 2 diabetes, combined aerobic exercise and a healthy diet has been associated with significant reductions in waist circumference (MD: −3.41; 95% CI: −6.00, −0.81), systolic (MD: −0.16; 95% CI: −0.30, −0.01) and diastolic blood pressure (MD: −0.19; 95% CI: −0.33, −0.06), and improvements in lipid levels [7].

Preliminary findings from IBD-specific studies suggest that adopting a prudent eating pattern or participating in physical activity may improve anthropometric cardiometabolic risk factors, without aggravating disease activity [6]. This highlights the potential of integrating preventative strategies, such as dietary and physical activity interventions, into IBD care to address both disease management and cardiometabolic risk.

However, most lifestyle research in IBD has primarily focused on inducing or maintaining remission, alleviating fatigue, and quality of life, with limited emphasis on cardiometabolic outcomes [6]. Thus, this study aims to investigate the effects of a personalised diet and physical activity intervention compared to usual care, primarily on body fat and secondarily on lean muscle mass, visceral adipose tissue, waist circumference, blood pressure, fasted lipid profile, C-reactive protein (CRP), faecal calprotectin, quality of life, dietary intake, and physical activity. It is hypothesised that personalised diet and physical activity will be more effective than usual care to improve body composition and other markers of cardiometabolic disease.

## 2. Materials and Methods

### 2.1. Study Design

The IBD Lifestyle, Food and Exercise (IBDLiFE) study was a randomised controlled trial investigating a personalised diet and physical activity intervention compared to usual care in adults with IBD in remission. This trial was approved by the Health and Disability Committee Northern B (reference: 2022 EXP 13602) and was registered at the Australian New Zealand Clinical Trials Registry (reference: ACTRN12622001417774, 7 November 2022). The study followed the CONSORT guidelines.

This study was conducted in accordance with the approved data and tissue management plan and institutional data policies. All participants provided informed consent for the collection and use of their data and blood specimens for this study. Data were collected primarily by the study investigator and the designated staff (phlebotomist and DXA technician), all trained in study protocol and collection requirements. Participant medical records were reviewed by the study investigator and entered into REDCap (Research Electronic Data Capture, V13), a secure web-based survey application, according to their unique identifiers. All survey data were stored in the University of Otago data centres, and all electronic data were kept in a password-protected computer located in the hospital.

Potential participants (*n* = 777 patients) from the NZ patient database of Crohn’s Colitis Care (CCCare), maintained by Crohn’s Colitis (CCCure), Australia, were contacted via text messages containing key study details between 2023 and 2024 by the research investigator. Additional recruitment took place in-person at the Dunedin hospital Medical Infusion Unit, through referrals from IBD nurses and gastroenterologists, and via self-referral from posters displayed in hospital and on local IBD support group social media platforms. Interested individuals completed the screening questionnaire online, and all participants provided written consent electronically prior to data collection.

Stratified randomisation was used to assign participants into the intervention group (personalised advice) and control group (usual care) based on IBD diagnosis. The random allocation sequence was computer-generated by the research biostatistician and uploaded onto REDCap (Nashville, Tennessee, Vanderbilt University). The research investigator was blinded to the randomisation list, but blinding was not possible for some of the measures. Blinding was maintained for the Dual-energy X-ray Absorptiometry (DXA) scans (primary outcome) and laboratory results (secondary outcomes), as these were measured by independent personnel. Participants were blinded to their group allocation.

Participants attended a one-hour study appointment and completed study outcome questionnaires at both baseline and the endpoint (month 6). All participants had five monthly phone calls (month 2, 3, 4, 5, 6) with the research investigator. Calls averaged 15 min for the intervention group (covering dietary topics and physical activity reviews) and approximately 3 min for the control group (maintaining engagement without additional input).

### 2.2. Study Population

Adults aged 18 years and above and diagnosed with IBD with stable medical therapy for at least 2 months and a Body Mass Index (BMI) > 25 kg/m^2^ were eligible for inclusion. Participants must not have severely active IBD defined as Harvey-Bradshaw index (HBI) ≤ 16 for those with Crohn’s disease (CD) and simple clinical colitis activity index (SCCAI) < 12 for those with ulcerative colitis (UC). Pregnant or breastfeeding women were not included. Participants were also excluded if they were on a liquid diet (e.g., exclusive enteral nutrition) or parenteral nutrition. Moreover, participants were excluded if they had a surgery in the last month or had an IBD-related surgery scheduled within the next 6 months. Participants with medical implants such as defibrillators or pacemakers or who had a body weight of more than 205 kg (weight limit for DXA scanner) were excluded. Participants were also ineligible to participate if they had a medical condition preventing participation in regular physical activity (assessed by Physical Activity Readiness Questionnaire) [8] or consumed a higher fibre diet (assessed by Dietary Fibre Intake-Food Frequency Questionnaire >25 g/day) [9]. Participants were enrolled at Dunedin, Dunstan, and Southland hospitals, in NZ, between 2023 and 2024 by the investigator.

### 2.3. Lifestyle Intervention

The intervention group received dietitian-led dietary education based on the Heart Foundation’s Heart Healthy Eating booklet [10]. This was personalised using the booklet’s dietary goal sheet following each of the monthly topics, including (1) fruits and vegetables, (2) wholegrain, (3) protein foods and dairy products, (4) dietary fats, and (5) discretionary food and drinks. Dietary goals were set using the SMART framework (Specific, Measurable, Achievable, Realistic, and Time-bound) [10]. For example, a SMART goal might be as follows: “Consume two servings of fruit per day by eating one serving (e.g., a banana or a handful of berries) in the morning and one serving (e.g., an apple or pear) in the afternoon”. To support participants and improve confidence in meeting their dietary goals for fruits and vegetables [11], a seasonal produce box of fruits and vegetables, consisting of half of the recommended weekly intake of fruits and vegetables, was delivered for the first 4 weeks of the intervention.

Participants also received a physical activity programme consisting of a booklet and a planner that was previously developed and trialled in a cohort of patients with IBD in Dunedin [12]. The programme encouraged participation in physical activity at least five times a week for a minimum of 10 min per session, with the goal of progressing towards the recommended guideline of 150 min of moderate-vigorous physical activity per week [13]. An activity planner was provided to help track progress towards personal exercise goals. Participants were asked to place a gold star sticker for each completed activity to promote positive reinforcement. Each week, participants reviewed their progress based on the number of stars earned and adjusted their exercise intensity or duration as needed, following the booklet. The self-directed nature of the programme was designed to promote autonomy and foster intrinsic motivation, both of which are associated with long-term exercise adherence [14]. This approach also reflects real-world challenges that are not captured in supervised interventions.

The control group received only usual care (generic diet and physical activity recommendations). Dietary resources included the *Eat for Health* pamphlet, *Simple Steps for Heart Healthy Eating* and *Mix & Match Snack Ideas*, developed by the NZ Heart Foundation, and were provided without personalisation to their current dietary intake. Similarly, the NZ Heart Foundation Physical Activity Guidelines were provided without personalisation.

During the monthly follow-up sessions, the intervention group received phone calls that included specific dietary topics from the provided resource, with goal setting and prompts for self-evaluating their physical activity progress. In the control group, phone calls were made to maintain engagement throughout the study, but no personalisation was provided.

### 2.4. Study Endpoints

All questionnaires were administered online via REDCap, and clinical outcomes were measured at the local hospitals at both baseline and endpoint (month 6).

### 2.5. Assessment of Participant Characteristics

Demographics and health questionnaire data were collected electronically using REDCap. Participants self-reported age, sex, ethnicity, IBD phenotype (CD or UC), previous IBD surgeries, presence of other co-morbidities, complementary and alternative medicines or supplements, prescribed medications, smoking status, and alcohol intake. The study investigator extracted the year of IBD diagnosis and location of disease from the Dunedin hospital and CCCare patient database. Any changes in medical therapies, smoking status, and alcohol consumption were recorded at endpoint.

### 2.6. Assessment of Primary Outcome

The primary outcome was change in body fat mass (kg) at endpoint, measured using a Lunar iDXA (GE Healthcare, Madison, WI, USA). The scan was performed by a trained technologist following manufacturer guidelines, with participants positioned supine and aligned to the central axis, arms placed slightly apart from the torso, and feet positioned to prevent movement. For participants who did not fit within the scanning field, “offset scanning” was conducted according to the International Society for Clinical Densitometry recommendation [15]. Scans were analysed using Lunar Encore software (version 18; GE Healthcare, Madison, WI, USA), and the DXA lab’s coefficients for variation for repeat in vivo scans were 1.8% for total fat mass, 1.8% for percentage fat, and 1.0% for bone-free lean tissue mass. Due to lack of DXA access, participants from Dunstan and Southland hospitals had body composition measured using bioimpedance analysis (BIA) (Bodystat 1500 MDD, Bodystat Ltd, Douglas, Ise of Man, British Isles). While BIA tends to overestimate muscle mass and underestimate fat mass in overweight or obese individuals [16], it can be used to accurately measure body composition changes over time with minimal differences compared to DXA [17]. To improve BIA accuracy, participants were instructed to avoid vigorous activity for 24 h, limit caffeine intake, and empty their bladder before measurement to control for hydration-related fluctuations.

### 2.7. Assessment of Clinical Outcomes

The secondary clinical outcomes included changes from baseline to endpoint between groups in other body composition parameters (lean muscle mass and visceral adipose tissue), waist circumference, and seated blood pressure measured following standard procedures [18]. Fasted lipid profile, C-reactive protein (CRP), and faecal calprotectin were collected at the Awanui Labs, Dunedin hospital. Clinical disease activity was self-reported using the HBI for CD and the SCCAI for UC [19].

### 2.8. Assessment of Diet and Physical Activity

Dietary intake data were collected using 3-day non-consecutive food diaries that included time of eating, food brands, portion sizes, and cooking methods. Each diary was reviewed with prompts and checked for completeness by the study investigator with the participant. The International Physical Activity Questionnaire Short Form (IPAQ-SF) was used to capture moderate- and vigorous-intensity activities, including activities such as walking and sitting [20]. IBD-related barriers to physical activity were measured in a multiple-choice question where participants could select more than one of the following options: “fatigue”, “muscle weakness”, “abdominal pain”, “joint pain”, “bowel incontinence”, “embarrassment related to symptoms”, and “others” [12].

### 2.9. Assessment of Quality of Life

Quality of life was assessed with the short IBD questionnaire (SIBDQ), consisting of 10 questions scored from 1 (worst of health) to 7 (best of health) [21]. The sum score ranges from 7 to 70, with higher scores representing better quality of life [21]. The Food Related Quality of Life-29 (FR-QoL-29) measures the psychosocial aspects of eating and drinking using 29 items rated on a five-point Likert scale (1 = strongly agree to 5 = strongly disagree) [22]. The sum score ranges from 29 to 145, with higher scores representing better food-related quality of life [22].

### 2.10. Adherence to Intervention

Adherence to the dietary intervention was assessed as the number of dietary education follow-up phone calls provided. A total of five follow-up phone calls were scheduled, with 100% adherence indicating the completion of all five sessions. Adherence to the physical activity intervention was assessed as the number of sessions completed over 6 months, with five sessions per week (130 sessions in total) indicating full adherence (100%).

### 2.11. Statistical Analysis

Specifying a two-sided alpha level of 0.05 (Type I error) and assuming equal standard deviations (SDs) across groups, a total sample of 64 (after an expected dropout rate of 20%), would ensure over 80% power to detect a 0.75 SD difference (medium-large effect size) in change in body fat mass between intervention and control groups. The recruitment sample size target was 80 participants due to practicality and feasibility (time, financial, and resource constraints).

Dietary data obtained from food diaries were entered into FoodWorks v.10.0 (Xyris Pty Ltd., Brisbane, Australia) for macro- and micronutrient analysis. Entries were reviewed for overall energy consumption and cross-checked against the food diaries to check the number of food items and any substitutions. Food items were coded into the food groups (1) vegetables, (2) fruits, (3) grain foods, (4) dairy and dairy alternatives, (5) meat, poultry, and seafood, and (6) legumes and lentils, according to the NZ eating guidelines [13] using Microsoft Excel (Microsoft corporation, Office 16, Redmond, WA, USA). Discretionary food and drinks were classified based on the Australian dietary guidelines, as NZ has yet to provide clear definitions and portion sizes for these foods [23]. Micronutrient supplement use was noted, but only fibre supplements were included in the analysis, as dietary fibre was a key outcome of interest. Physical activity data from the IPAQ-SF were converted to continuous metabolic equivalent task (MET) minutes/week and further categorised into low, moderate, and high according to its scoring protocol.

Continuous variables were presented as either means and SD or median (lower quartile (LQ), upper quartile (UQ)) based on their distribution, or categorised into relevant groups. Categorical variables were described as frequencies and percentages. These variables were stratified by the group assignment (intervention vs. control). Associations between the groups and each outcome measure were investigated using a multiple linear regression model and included the respective baseline value as a covariate. All models were adjusted for both age and sex, and additional potential confounders were incorporated based on clinical context. For example, blood pressure and lipid profile were adjusted for the use of relevant medications (blood pressure or lipid-lowering medications), diagnoses of hypertension or high cholesterol, and smoking status; dietary and physical activity data were adjusted for seasonality and clinical disease activity; quality of life was adjusted for clinical disease activity and barriers to physical activity, and FR-QoL-29 was adjusted for clinical disease activity. The residuals of the linear regression models were assessed for concerns regarding assumptions (heteroscedasticity and non-normal distribution). Statistical analysis was performed in Stata version 18.0 (StataCorp, College Station, TX, USA, 2024).

The regression coefficient refers to the difference in the changes of the outcome between the groups. Specifically, a positive coefficient could indicate that the intervention group had either a greater increase or a smaller decrease (across two timepoints) in the measured outcome compared to the control group. A negative coefficient suggests the opposite, indicating either a smaller increase or a greater decrease, correspondingly.

## 3. Results

### 3.1. Participant Flow Diagram

**Figure 1:**
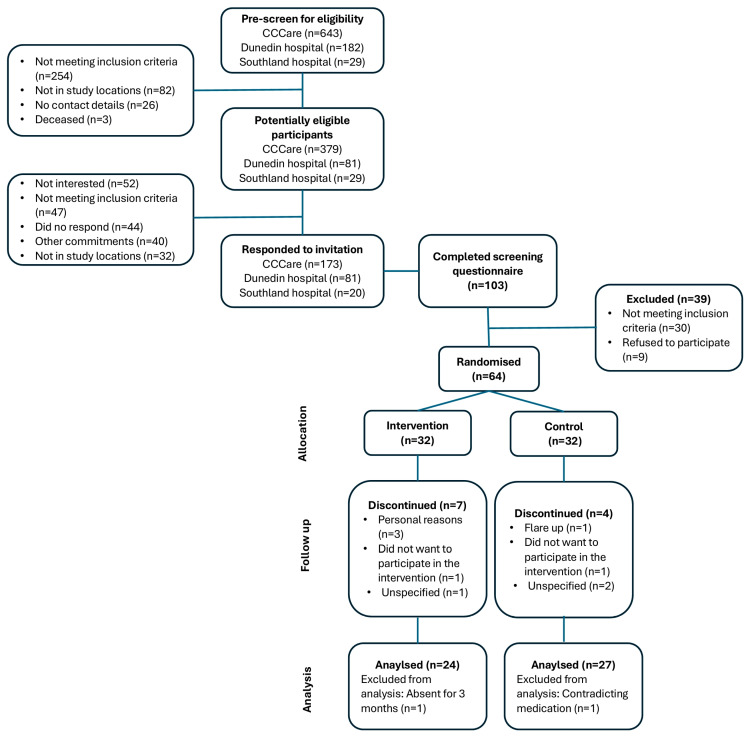
CONSORT flow diagram of the IBDLiFE study.

Among the 489 potentially eligible participants, 274 responded, and 103 completed the screening process. Thirty participants were then excluded for not meeting the inclusion criteria: 13 had a dietary fibre intake >25 g/day, six had a BMI < 25 kg/m^2^, four had both high dietary intake and low BMI, four were unable to participate in physical activity, two were scheduled for surgery, and one was receiving nutritional support. After randomisation, eight participants formally withdrew from the study with reasons shown in Figure 1, and three were lost to follow-up. Two participants were subsequently excluded from the analyses: one for undisclosed use of liraglutide (Saxenda^®^) for weight loss during the eligibility phase, and the other for unreported travel plans for 3 months prior to randomisation. Ultimately, 51 participants completed the study. The overall attrition rate of the study was 17.7%, with dropout rates of 22.6% in the intervention group and 12.9% in the control group (difference: 9.7%; 95% CI: −9.2%, 28.6%; *p* = 0.318).

### 3.2. Participant Demographics and Health Characteristics

Participant demographics and clinical characteristics are summarised in Table 1. Demographic characteristics were similar between the two groups except for sex, where there was a higher proportion of females in the intervention group than in the control group (68.7% vs. 50.0%). The median age of the cohort was 47 years (LQ: 33, UQ: 55), and the majority identified as NZ European ethnicity (90.6%). Most participants had never smoked (79.7%). The most common cardiometabolic risk factor was high waist circumference (87.5%), followed by abnormal lipid profile and elevated blood pressure. Nearly half of participants reported other medical conditions. There were more participants with CD (60.9%) than UC (39.1%), and the most common maintenance therapy was biologics (43.8%), while 18.8% were not on medication. A lower proportion of participants from the intervention group compared to the control group were in clinical disease remission (28.1% vs. 59.4%). However, faecal calprotectin levels suggested that 85% of the cohort were in biological remission.

The baseline characteristics of participants who completed the study and those who dropped out (23% in the intervention group, *n* = 7; and 13% in the control group, *n* = 4) are available in Supplementary Table S1.

### 3.3. Clinical Outcomes

Table 2 presents the difference between groups for clinical outcomes, including body composition, waist circumference, blood pressure, lipid profile, and inflammatory biomarkers. There were no statistically significant differences in body fat between the two groups (−0.2 kg; 95% CI: −1.6, 1.2). Similarly, no significant differences were observed between the groups for any of the clinical outcomes. However, the intervention group demonstrated a non-significant tendency towards a lower change in the total/high-density lipoprotein-cholesterol ratio (−0.16; 95% CI: −0.42, 0.09) compared to the control group. This was concurrent with a non-significant tendency towards a more positive change in high-density lipoprotein-cholesterol (0.07 mmol/L; 95% CI: −0.03, 0.17).

### 3.4. Dietary Intake

Table 3 presents the difference between groups for total intakes of energy, macro- and micronutrients, and food groups. The intervention group significantly increased their fibre intake (3.1 g/1000 kcal; 95% CI: 1.1, 5.1) and decreased their sodium intake (−911.2 mg/day; 95% CI: −1782.8, −39.6) compared to the control group. These dietary intakes aligned with significantly greater intakes of fruit (0.5 serves/day; 95% CI: 0.1, 1.0) and lower intakes of discretionary food and drink (−1.7 serves/day; 95% CI: −3.0, −0.3). Although not statistically significant, there was also a tendency towards a lower change in total energy intake, as well as a higher change in both protein and total fibre intake (g/day).

### 3.5. Physical Activity and Quality of Life

**Figure 2:**
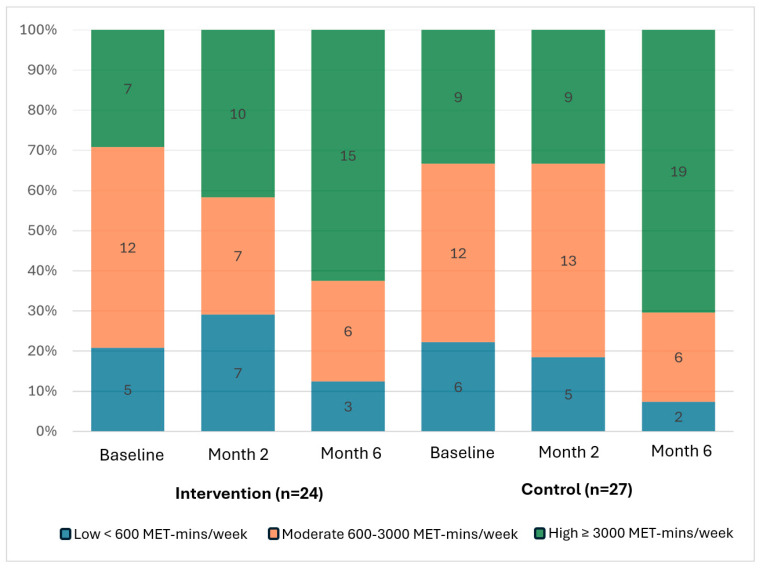
Proportion of participants with complete data in each physical activity intensity category at each timepoint for the intervention and control group.

Table 4 presents the difference between groups for physical activity and quality of life. Overall, there were no statistically significant differences in changes across all intensities of physical activity or time spent sitting, as well as quality of life between the two groups. However, exploratory within-group descriptive analyses suggested that the proportion of highly active participants increased from baseline to month 6 in both groups (Figure 2). This was accompanied by an overall general pattern of decrease in the proportion of participants reporting barriers to physical activity in both groups.

### 3.6. Intervention Adherence

The intervention group fully adhered to the dietary intervention with five complete follow-up sessions, while the average adherence to the physical activity intervention was 71.4%. Nearly one-third of patients (29%, *n* = 7) achieved full adherence, completing all 130 physical activity sessions, while 29% (*n* = 7) completed less than 50% of sessions.

## 4. Discussion

People with IBD are more likely to develop CVD due to systemic inflammation [24] and have a higher prevalence of cardiometabolic risk factors compared to the general population [5], which may be modified through diet and physical activity [4]. Non-IBD studies with larger samples have reported significant changes in body composition [25,26], typically through calorie-restricted diets [25] and supervised or individualised exercise interventions [26]. While effective, such approaches may be unsustainable and less suitable for individuals with IBD due to their restrictive nature and disease-specific challenges with diet and physical activity [6,27,28]. Recognising this, our study adopted a more flexible, real-world approach to support healthy eating and an active lifestyle. In this randomised controlled trial, we evaluated a personalised intervention integrating heart-healthy dietary guidance with a self-directed physical activity programme against usual care, to improve body composition and other markers of cardiometabolic disease.

We demonstrated that personalised dietary education led to a significant increase in fibre intake in the intervention group compared to the control group at month 6. A 3 g/1000 kcal/day change in dietary fibre intake is equivalent to consuming a medium piece of fruit (e.g., banana, orange, or apple), 1/3 cup of frozen vegetables, or a slice of wholegrain bread. This improvement was likely driven by a significant increase in fruit consumption, with intervention participants consuming half a serving more per day than the usual care group. While not statistically significant, there was also a positive increase in legume and lentil intake (another source of dietary fibre). Additionally, dietary education for the intervention group emphasised substituting refined grains with wholegrains, such as switching from white to brown bread, which may have further contributed to the increased fibre intake. These findings are comparable to other IBD studies showing that various personalised approaches, whether through online pre-formatted advice based on a scoring system [29] or in-person counselling with a dietitian [30,31], enhance adherence to incorporating more fibre-rich foods. This is especially relevant given that up to 43% of people with IBD in remission avoid fibre-rich foods often due to the misconception that fibre exacerbates gut symptoms [27]. Consequently, average fibre intake among patients with quiescent IBD tends to be lower than that of the general population, with reported intakes averaging 16 g/day (SD:6) [32]. This is well below the recommended 25–30 g/day for the prevention of diabetes and CVD [13].

In addition to increased dietary fibre intake, the intervention group also significantly reduced consumption of discretionary foods and sodium compared to the control group, although no significant difference was observed in total energy intake. This may be explained by a modest increase in physical activity within both groups, which may improve appetite [33], potentially offsetting reductions in discretionary food intake. Nonetheless, our findings align with the findings of the Food4Me study, a cohort of 1607 adults without IBD across seven European countries [34]. This study showed that personalised online nutrition advice led to significantly lower percentages of energy, fat, saturated fatty acids, and salt from discretionary foods compared to those who did not receive the intervention [34]. Similarly, a small uncontrolled trial involving 26 people with IBD who received 6 months of dietitian-led counselling reported improvements in adherence to dietary guidelines, including reduced consumption of processed foods and increased intake of vegetables, fruits, legumes, and nuts [31].

Collectively, these findings, including our study, demonstrated the potential of personalised dietary education to encourage healthier eating habits, even among individuals with IBD who may face multiple perceived barriers to dietary changes. The dietary improvements observed in our study, namely reduced discretionary food and increased dietary fibre consumption, are particularly meaningful given that people with IBD tend to consume more discretionary foods such as confectionaries and sugar-sweetened beverages than the general population [32,35]. A meta-analysis also found higher intake of added sugars and lower intake of vegetables, fruits, and wholegrains among individuals with IBD, suggesting a shift away from whole foods toward nutrient-poor discretionary options [32]. This pattern is concerning, as high intake of energy-dense, nutrient-poor discretionary foods, often rich in salt, sugar, or alcohol, is associated with increased risk of obesity, diabetes, and CVD. In contrast, high fibre diets are not only cardioprotective [4], but may also play a role in IBD management. For instance, greater fibre intake has been associated with a lower risk of CD relapse [36]. This may be attributed to the increased diversity of gut microbiota, such as *Bifidobacterium* spp., *Lactobacilllus* spp., and *Faecalbacterium prausnitzii*, which produce short-chain fatty acids [37]. These microbial metabolites, namely butyrate, acetate, and propionate, are known to regulate metabolic and immune homeostasis and maintain gut barrier integrity [38]. Moreover, a recent review on functional dietary fibre and irritable bowel syndrome suggests that strategies involving the personalisation of dietary fibre based on their proposed mechanisms (bulking, viscous, and fermentation) may help modulate GI symptoms [39]. This highlights the potential for utilising dietary fibre interventions to improve IBD symptoms; however, a more comprehensive understanding of their underlying mechanisms, along with higher-quality clinical evidence, is needed to fully assess their therapeutic value and relevance in IBD management. Nevertheless, our findings, consistent with the existing literature, showed that adherence to a healthy diet [29,31] or increasing fibre intake [30] during remission did not negatively affect disease activity, reinforcing the potential of this approach for people with IBD.

Given that food avoidance in IBD can negatively impact quality of life, we anticipated a significant difference in quality of life between the two groups. However, although the personalised group avoided foods less often at month 6, there were no significant differences in quality of life between the two groups. This contrasts with previous research suggesting that a less restrictive diet is associated with better FR-QoL in people with IBD [40] and that a more healthful diet is also associated with greater IBD quality of life [41]. The lack of observed differences between the groups in our study could be attributed to a placebo effect, as the control group also received generic dietary education and monthly follow-up phone calls. Additionally, both groups showed an increase in quality of life over the six-month period. It is also possible that the higher baseline FR-QoL scores in our study (mean score: 95), compared to previous research (mean score: 81) [41], meant there was limited scope for further improvement.

Overall, the intervention group reported several positive changes in diet, whereas physical activity did not differ significantly between the groups. This could be attributed to the variability in participants’ adherence to the physical activity intervention, as approximately one-third of participants completed less than 50% of the recommended sessions. This suggests that some participants may still struggle to incorporate physical activity into their daily routines. It is unclear whether these challenges are solely due to IBD-related barriers or whether they were compounded by common barriers such as lack of motivation, competing commitments, limited access to facilities, or time constraints. However, exploratory analyses suggested that the proportion of participants reporting IBD-related physical activity barriers decreased over the course of the study for both groups, while physical activity level increased. This may suggest that adherence is influenced by factors beyond IBD alone. For instance, seasonal variation could play a role, as 60% of participants were recruited during autumn and winter, meaning their six-month follow-up was in spring or summer, when physical activity is typically more feasible and appealing [42].

There were no significant differences in body composition observed between the intervention and control groups. This result is difficult to compare with previous diet or physical activity IBD studies, as most research that has assessed change in body composition involves small sample sizes (n < 25) [43,44], lacks a control group [3,44], is of short duration [43,44], or did not measure body composition as the primary outcome [44]. Similarly to our findings, an uncontrolled dietary trial involving a 6-month isocaloric Mediterranean diet had no effect on body composition [3]. On the contrary, collective evidence from small crossover and uncontrolled supervised physical activity studies demonstrated reductions in body fat of up to 2.1%, following prescribed exercise [43,44]. The lack of significant changes in body composition in our study may be attributed to the absence of differences in physical activity intensities and volume, as well as overall energy intake between groups.

The key strength of this study is its randomised controlled design, which minimises selection bias and facilitates valid comparisons between the intervention and control groups. Most body composition measurements were assessed using the gold standard method, DXA. Dietary intake was measured using three non-consecutive day food diaries, including two weekdays and one weekend, providing a comprehensive and detailed record of participants’ intake. This allowed the research dietitian to offer personalised advice tailored to each participant’s habits and preferences. Another major advantage is that the combined diet and physical activity intervention involved a multidisciplinary team approach comprising a gastroenterologist, dietitians, a sport and exercise physician, a body composition expert, and a biostatistician. This ensured a well-rounded and expert-informed approach. Another strength of the intervention was the personalised education provided by the research dietitian, particularly regarding diet.

This study has several limitations. Firstly, as it was a single blinded intervention delivered by the investigator, blinding was not possible for some data collection (e.g., anthropometrics, blood pressure, food diaries, and physical activity diaries). However, the primary outcome assessment (DXA scans) was conducted by a technician blinded to the study intervention allocation, and secondary biomarker outcomes were independently collected and analysed by the hospital laboratories. Secondly, self-reported physical activity is susceptible to recall and social desirability biases which may lead to over- or under-reporting of activity levels. Unfortunately, objective measurement was not feasible due to resource constraints. Although the produce box provided to the intervention group may also introduce bias by enhancing perceived benefits and motivation, the short duration (4 weeks) likely had minimal impact on the six-month outcome. Responder bias may be present, but demographic similarities with previous regional study [45] support generalisability to the broader NZ population with inactive IBD. Lastly, the design of the control group may potentially underestimate the intervention effects, as several outcomes in the control group, such as quality of life and physical activity, also improved. These may reflect the influence of monthly investigator contacts, which, though intended to isolate the intervention effect, may have provided unintended benefit.

Future IBD research may benefit from strategies designed to elicit greater differences in physical activity levels, as small (n < 25), short-term (<12 weeks), uncontrolled studies involving supervised physical activity have shown reductions in body fat of up to 2.1% [6]. This could be achieved by considering the intensity and type of activity, given that different exercise modalities affect body composition differently. For example, resistance activities better promote gains in lean body mass [13]. Exercise programmes should also be led by qualified professionals, such as sports and exercise physicians or physiotherapists [46], to address specific barriers (e.g., arthritis-related joint pain) and provide tailored guidance for this population. This approach would allow for better personalisation of the intervention within an interdisciplinary model. Accounting for differences in energy intake may help initiate body composition changes; however, this requires careful consideration given the high prevalence of food restrictions [47] and disordered eating patterns in those with IBD [48] associated with psychological distress [40]. Dietary interventions should continue to focus on the principles of healthy eating to ensure nutritional adequacy and improve diet variety with personalisation delivered by a dietitian [30,31], as demonstrated to be feasible within this study. Moreover, future studies could incorporate objective measures of diet and physical activity to minimise bias from self-report. For example, accelerometers could provide detailed data on activity intensity, frequency, and duration, while cardiopulmonary exercise testing (VO_2max_) can better quantify changes in cardiometabolic risk. Dietary biomarkers such as serum cholesterol or metabolomics-based approaches [49] may further strengthen dietary assessment, though challenges remain in identifying and annotating the vast number of food-derived metabolites [50]. This highlights the need for continued advances in computational analysis to interpret complex metabolomic data to support precise dietary changes in the future. The use of speckle tracking echocardiography, where expertise and resources permit, may also be valuable for detecting subclinical cardiovascular changes to evaluate the effectiveness of lifestyle interventions and provide mechanistic insights [51]. Finally, including a minimal contact control group in future trials would help determine the independent effects of personalisation, as brief monthly contact in our study appeared sufficient to improve physical activity and quality of life, suggesting a potentially scalable and cost-effective model for clinical practice. All in all, future lifestyle interventions should incorporate personalised dietary support from dietitians, alongside input from sport and exercise physicians or physiotherapists, to facilitate the synergistic effects of diet and physical activity on body composition in a population with IBD.

## 5. Conclusions

A personalised education improved dietary fibre intake and reduced sodium intake, alongside significant increases in fruit and reductions in discretionary food consumption. These changes suggest that such an approach may improve participants’ confidence in trying new foods or reintroducing previously avoided foods without aggravating disease activity, highlighting the value of dietitian support.

## Figures and Tables

**Table 1:** Baseline demographics and health characteristics of 64 patients with inflammatory bowel disease.

	Intervention (*n* = 32)	Control (*n* = 32)	Total (*n* = 64)
Age, median (LQ, UQ)	46 (41, 53)	51 (34, 56)	47 (37, 55)
Biological sex—Female, *n* (%)	22 (68.7)	16 (50.0)	38 (59.4)
Ethnicity, *n* (%) ^1^			
NZ European	29 (90.6)	29 (90.6)	58 (90.6)
Māori	1 (3.1)	3 (9.4)	4 (6.3)
Others	3 (9.4)	3 (9.4)	6 (9.4)
Smoking status, *n* (%)			
Active	1 (3.1)	1 (3.1)	2 (3.1)
Ex-smoker	6 (18.8)	5 (15.6)	11 (17.2)
Vaping status- Yes, *n* (%) ^2^	2 (6.3)	2 (6.3)	4 (6.3)
Cardiometabolic risk factors ^3^			
BMI, mean (SD)	32.6 (5.4)	29.7 (4.5)	31.2 (5.2)
High waist circumference, *n* (%)	29 (90.6)	27 (84.4)	56 (87.5)
Lipid abnormalities. *n* (%)	18 (56.3)	18 (56.3)	36 (56.3)
Elevated blood pressure, *n* (%)	6 (18.8)	3 (9.4)	9 (14.1)
Comorbidities—Yes, *n* (%)	14 (43.8)	16 (50.0)	30 (46.9)
High blood pressure	4 (12.5)	6 (18.8)	10 (15.6)
Diabetes	1 (3.1)	1 (3.1)	2 (3.1)
High cholesterol	1 (3.1)	1 (3.1)	2 (3.1)
Asthma	4 (12.5)	4 (12.5)	8 (12.5)
Arthritis	2 (6.3)	1 (3.1)	3 (4.7)
Others	5 (15.6)	5 (15.6)	10 (15.6)
IBD measures			
Disease duration, median (LQ, UQ)	13 (7, 20)	11 (7, 16)	12 (7, 19)
Crohn’s disease, *n* (%)	17 (53.1)	22 (68.8)	39 (60.9)
Ileal	3 (17.7)	5 (22.7)	9 (23.1)
Colonic	3 (17.7)	6 (27.3)	8 (20.5)
Ileocolonic	11 (64.7)	11 (50.0)	22 (56.4)
Upper GI	0 (0)	0 (0)	0 (0)
Ulcerative colitis, *n* (%)	15 (46.9)	10 (31.3)	25 (39.1)
Proctitis	2 (13.3)	1 (10.0)	3 (12.0)
Left sided	6 (40.0)	6 (60.0)	12 (48.0)
Extensive	7 (46.7)	3 (30.0)	10 (40.0)
IBD resections—Yes, *n* (%)	9 (28.1)	5 (15.6)	14 (21.9)
Maintenance therapy, *n* (%)			
None	6 (18.8)	6 (18.8)	12 (18.8)
Aminosalicylates	10 (31.3)	7 (21.9)	17 (26.6)
Immunomodulators	10 (31.3)	14 (43.8)	24 (37.5)
Biologics	14 (43.8)	14 (43.8)	28 (43.8)
Disease activity, *n* (%) ^4^			
Remission	9 (28.1)	19 (59.4)	28 (43.8)
Mild	18 (56.3)	10 (31.3)	28 (43.8)
Active	5 (15.6)	3 (9.4)	8 (12.5)
Inflammatory biomarkers, *n* (%)			
Faecal calprotectin ^5^			
Remission, <150 µg/g	23 (79.3)	28 (90.3)	51 (85.0)
Mild, 150–250 µg/g	2 (6.9)	1 (3.2)	3 (5.0)
Active, >250 µg/g	4 (13.8)	2 (6.5)	6 (10.0)
CRP			
<5 g/L	23 (71.9)	26 (81.3)	49 (76.6)
>5 g/L	9 (28.1)	6 (18.8)	15 (23.4)

^1^ Self-identified ethnicity was classified into three ethnic groups using the 2006 NZ census ethnicity questions and the Ministry of Health classification system. Participants could select multiple ethnicities, so column totals do not necessarily total 100%. Other ethnicities included British, European, Dutch (intervention group) and German, Scottish, European (control group). ^2^ Vape with nicotine content. ^3^ Cardiometabolic risk factors include high waist circumference: >94 cm (male); >80 cm (female); elevated blood pressure: >140 mmHg systolic and/or >90 mmHg diastolic; abnormal lipid profile: high LDL cholesterol > 3.4 mmol/L, high triglyceride >2.0 mmol/L, high total cholesterol/HDL >4.5. ^4^ Disease activity was measured using the Harvey Bradshaw Index (HBI) for CD and the Simple Clinical Colitis Activity Index (SCCAI) for UC and IBD-unspecified. Disease activity was classified according to the following HBI scores: <5 remission, 5–7 mildly active, and ≥8 active 186; and SCCAI scores: ≤2 remission, <5 mildly active, and ≥5 active. ^5^ Total number of data points *n* = 60. Missing data from intervention (*n* = 3) and control group (*n* = 1). LQ = lower quartile; UQ = upper quartile; n = number; BMI = body mass index; NZ = new zealand; IBD = inflammatory bowel disease; GI = gastrointestine; CRP = C-reactive protein.

**Table 2:** Between-group differences in clinical outcome changes from baseline to month 6 among 51 patients with inflammatory bowel disease.

	Intervention		Control		Adjusted Mean Difference (95% CI)
	**Baseline** **(*n* = 31)**	**Month 6** **(*n* = 24)**	**Baseline** **(*n* = 31)**	**Month 6** **(*n* = 27)**	***n*** **= 51 ^1^**
**Body composition ^1,2^,** **median (LQ, UQ)**					
Fat mass, kg	38.3 (31.0, 45.7)	38.1 (32.4, 46.1)	28.3 (24.4, 36.2)	27.6(24.8, 36.2)	−0.2 (−1.6, 1.2)
Fat mass index (kg/m^2^)	13.6 (11.2, 16.5)	13.4 (11.3, 16.4)	9.9 (8.2, 12.8)	9.9 (8.4, 12.1)	−0.03 (−0.5, 0.5)
Body fat percentage	43.9 (37.4, 49.1)	43.6 (37.1, 48.0)	36.7 (31.1, 41.5)	36.9 (32.5, 42.7)	0.21 (−0.7, 1.2)
Lean mass, kg	47.1 (43.7, 54.5)	47.0 (43.2, 54.6)	50.7 (40.8, 61.0)	49.1 (42.0, 60.5)	−0.4 (−1.0, 0.2)
Lean mass index (kg/m^2^)	16.5 (15.6, 19.2)	16.6 (15.6, 18.9)	16.9 (15.3, 19.0)	16.8 (15.5, 18.9)	−0.10 (−0.3, 0.1)
Visceral adipose tissue **^2^**, g	1794 (1087, 2053)	1552 (1138, 2349)	1310 (445, 1950)	1100 (600, 1796)	46.7 (−60.2, 153.6)
**Waist circumference, cm median (LQ, UQ)**	107 (93.5, 111.6)	103.3 (95.9, 110.5)	97.3 (87.8, 105.8)	95.6 (83.3, 105.8)	−0.21 (−2.3, 1.9)
**Blood pressure ^3^, mmHg** **median (LQ, UQ)**					
Systolic	123 (117, 138)	123 (119, 130)	124 (117, 134)	121 (115, 130)	2.0 (−3, 7)
Diastolic	80 (77, 86)	82 (78, 86)	81 (74, 85)	78 (74, 85)	2.0 (−1, 5)
**Lipid profile ^4^ (mean, SD)**					
Total cholesterol, mmol/L	5.3 (1.0)	5.2 (0.9)	5.0 (1.0)	4.9 (1.0)	0.05 (−0.3, 0.4)
Triglycerides,	1.3 (0.5)	1.3 (0.5)	1.4 (0.8)	1.5 (0.9)	−0.07 (−0.3, 0.2)
LDL-cholesterol, mmol/L	3.3 (0.9)	3.2 (0.9)	3.0 (0.8)	2.9 (0.9)	−0.02 (−0.3, 0.2)
HDL-cholesterol, mmol/L	1.4 (0.3)	1.5 (0.4)	1.4 (0.3)	1.4 (0.3)	0.07 (−0.0, 0.2)
Total cholesterol/ HDL cholesterol ratio	3.9 (1.0)	3.7 (1.0)	3.6 (0.8)	3.6 (0.9)	−0.16 (−0.4, 0.1)
**Disease activity (median, LQ, UQ)**					
HBI	5 (3, 6)	5 (2, 5)	3 (1, 6)	5 (1, 7)	−0.5 (−2.6, 1.5)
SCCAI	4 (3, 5)	3 (2, 4)	2 (1, 3)	1 (0, 3)	0.5 (−1.1, 2.1)
**Inflammatory biomarkers (median, LQ, UQ)**					
CRP, g/L	3 (1, 7)	4 (2, 6)	2 (1, 3)	3 (1, 6)	−44.7 (−171, 81)
Faecal calprotectin ^5^, µg/g	18 (16, 110)	20 (16, 92)	44 (16, 107)	40 (16, 182)	−0.85 (−3, 1)

Abbreviations: *n* = number, HBI = Harvey Bradshaw Index, HDL = high-density lipoprotein, LDL = low-density lipoprotein, LQ = lower quartile, SCCAI = Simple Clinical Colitis Index, UQ = upper quartile, SD = standard deviation. Regression analysis only includes participants with complete data: Intervention group (*n* = 24), Control group (*n* = 27). ^1^ Body composition was measured using DXA (*n* = 52) and BIA (*n* = 10) at baseline; DXA (*n* = 44) and BIA (*n* = 8) at month 6. Visceral adipose tissue was measured using DXA only (*n* = 44). ^2^ Models were additionally adjusted for differences in measurement methods. ^3^ Models were additionally adjusted for lipid-lowering medication, a diagnosis of high cholesterol, and smoking habits. ^4^ Models were additionally adjusted for blood pressure-lowering medication, a diagnosis of hypertension, and smoking habits. ^5^ The total number of data points for faecal calprotectin *n* = 58 at baseline and *n* = 51 at month 6. CRP = c-reactive protein.

**Table 3:** Between-group differences in dietary intake changes from baseline to month 6 among 51 patients with inflammatory bowel disease.

	Intervention		Control		Adjusted Mean Difference (95% CI)
	**Baseline (*n* = 30) ^2^**	**Month 6 (*n* = 24)**	**Baseline (*n* = 31)**	**Month 6 (*n* = 27)**	**(*n* = 51)**
**Dietary intake (median, LQ, UQ)**					
Total energy, kJ/day	8365 (6142, 9207)	6435 (5749, 7996)	8796 (6197, 9849)	7144 (5863, 9371)	−753 (−1669, 163)
Total energy, kcal/day	1999 (1468, 2201)	1538 (1374, 1911)	2102 (1481, 2354)	1707.6 (1401, 2240)	−180 (−399, 39)
Protein (g/day)	79.6 (71.0, 89.7)	79.0 (68.8, 90.0)	84.8 (71.8, 110.0)	77.1 (65.5, 100.2)	2.3 (−8.4, 13.0)
Protein (%TE/day)	16.4 (14.7, 18.8)	20.1 (17.7, 23.0)	18.2 (14.6, 20.7)	17.9 (15.9, 21.2)	1.7 (−0.5, 4.0)
Total Fat (g/day)	78.5 (57.4, 98.4)	67.8 (51.6, 83.5)	82.9 (63.4, 110.6)	70.3 (50.5, 93.9)	−9.2 (−24.2, 5.7)
Fat (% total TE/day)	36.2 (33.0, 40.7)	35.9 (33.3, 43.2)	39.7 (34.4, 42.1)	36.0 (33.0, 40.8)	−0.7 (−5.0, 3.7)
Saturated fat (g/day)	28.9 (23.6, 42.6)	24.3 (17.9, 33.9)	31.2 (26.5, 44.1)	31.1 (19.5, 37.5)	−4.4 (−10.7, 1.9)
Saturated fat (% TE/day)	14.9 (11.3, 16.7)	13.1 (11.0, 16.4)	15.0 (13.3, 18.4)	14.2 (11.4, 15.9)	−0.6 (−2.8, 1.6)
Monounsaturated fat (g/day)	25.5 (20.5, 36.8)	22.3 (18.4, 31.5)	29.5 (20.8, 38.6)	24.5 (18.8, 34.2)	−4.5 (−11.1, 2.0)
Monounsaturated fat (% TE/day)	13.1 (10.9, 15.0)	13.3 (10.3, 15.4)	13.2 (11.4, 15.3)	12.7 (11.4, 11.6)	−0.8 (−3.0, 1.4)
Polyunsaturated fat (g/day)	9.6 (8.1, 11.8)	9.4 (7.2, 13.6)	10.6 (6.7, 14.6)	8.3 (6.9, 12.1)	−0.2 (−3.0, 2.7)
Polyunsaturated fat (% TE/day)	4.9 (3.9, 5.7)	5.1 (4.2, 7.0)	4.7 (3.5, 5.6))	4.7 (3.7, 6.0)	0.4 (−0.8, 1.7)
Carbohydrate (g/day)	203.0 (158.4, 232.1)	146.9 (118.3, 185.6)	187.9 (153.0, 231.8)	172.6 (145.4, 212.9)	−22.6 (−47.8, 2.6)
Carbohydrate (% TE/day)	40.9 (37.3, 46.2)	38.3 (34.7, 42.7)	40.9 (34.2, 43.8)	40.3 (35.2, 44.4)	−1.4 (−5.4, 2.6)
Sugars (g/day)	82.8 (57.9, 109.0)	67.2 (52.3, 92.4)	78.3 (58.4, 99.5)	77.6 (60.9, 94.7)	−4.9 (−20.0, 10.2)
Sugars (% TE/day)	17.7 (13.2, 21.0)	17.3 (12.8, 20.7)	16.0 (11.7, 19.7)	15.7 (13.8, 19.2)	0.8 (−2.8, 4.4)
Fibre (g/day)	18.8 (15.2, 24.0)	20.4 (17.5, 22.8)	20.1 (17.3, 25.9)	18.6 (14.1, 21.6)	3.3 (−0.1, 6.7)
Fibre (g/1000 kcal/day)	9.5 (8.3, 11.4)	12.6 (11.1, 14.2)	10.4 (7.9, 12.5)	9.6 (8.3, 12.8)	3.1 (1.1, 5.1) **
Sodium (mg/day)	2621 (1986, 3080)	2217 (189, 2617)	2671 (1986, 3259)	2567 (2079, 3972)	**−911** (−1783, −40) *
Calcium (mg/day)	775 (638, 926)	782 (683, 996)	902 (577, 1085)	825 (561, 979)	30 (−90, 151)
**Food groups ^1^, serves per day**					
Vegetables	2.9 (1.5, 3.9)	2.8 (2, 3.9)	2.7 (1.6, 3.9)	2.2 (1.5, 2.8)	0.7 (−0.4, 2.0)
Grains	2.9 (2.4, 4.1)	3.2 (1.9, 3.7)	3.6 (1.7, 4.7)	2.9 (2.6, 4.2)	−0.0 (−0.6, 0.7)
Fruits	0.6 (0.2, 1.0)	1.3 (0.5, 2.0)	0.8 (0, 1.4)	0.6 (0.1, 1.1)	0.5 (0.1, 1.0) *
Dairy and non-dairy products	1.6 (0.8, 2.5)	1.8 (1.0, 2.5)	1.7 (0.7, 2.6)	1.7 (1.0, 2.1)	0.0 (−0.4, 0.5)
Meats, poultry, seafood	1.5 (1.2, 2.1)	1.6 (1.2, 2.3)	1.9 (1.1, 2.6)	1.5 (0.8, 2.5)	0.1 (−0.4, 0.7)
Legumes and lentils	0.1 (0, 0.7)	0.1 (0, 1.3)	0.2 (0, 1.0)	0.1 (0, 0.6)	0.2 (−0.1, 0.5)
Discretionary food and drinks	4.8 (3.2, 7.5)	2.9 (1.3, 4.4)	4.3 (2.8, 6.7)	4.6 (2.8, 6.0)	−1.7 (−3.0, −0.3) *

Abbreviations: kcal = kilocalories, kJ = kilojoule, LQ = lower quartile, TE = total energy intake, UQ = upper quartile. Separate regression models were used for each outcome, with baseline values included as a covariate and adjustments made for age, sex, season, and disease activity. Regression analysis only includes participants with complete data: Intervention group (*n* = 24), Control group (*n* = 27). ^1^ Discretionary food include alcohol (beer, wine, spirits), high fat/sugary/salty spreads, commercial lasagna, baking, pastries, fries, quiche, sausage rolls, sugar-sweetened beverages, and confectionaries. ^2^ One patient did not provide a baseline food diary. * *p* <0.05; ** *p* < 0.01.

**Table 4:** Between-group differences in physical activity and quality of life changes from baseline to month 6 among 51 patients with inflammatory bowel disease.

	Intervention		Control		Adjusted Mean Difference (95% CI)
	**Baseline (*n* = 31)**	**Month 6 (*n* = 24)**	**Baseline (*n* = 31)**	**Month 6 (*n* = 27)**	**(*n* = 51)**
**Physical activity** ^1^					
**MET, mins/week**					
Walking	1188 (297, 1617)	2080 (840, 4680)	462 (198, 1188)	1440 (480, 2880)	543 (−1045, 2131)
Moderate	594 (198, 1188)	1680 (280, 4080)	594 (297, 1980)	1920 (1080, 3360)	−382 (−3386, 2622)
Vigorous	0 (0, 594)	60 (0, 1320)	0 (0,495)	0 (0, 1920)	311 (−707, 1330)
Total	1782 (990, 4108)	5260 (2140, 14,400)	1287 (891, 3465)	4800 (3200, 10,440)	188 (−3336, 3711)
**Time spent sitting, hours/day**	5.0 (4.0, 9.0)	5.5 (3.5, 8.0)	5.0 (3.0, 9.0)	5.0 (3.0, 8.0)	0.1 (−1.5, 1.8)
**Perceived barriers to physical activity-Yes, n (%)**	15 (48.4)	5 (20.8)	10 (32.3)	5 (18.5)	
Fatigue	13 (41.9)	5 (20.8)	9 (29.0)	5 (18.5)	
Muscle weakness	2 (6.5)	1 (4.2)	5 (16.1)	2 (7.4)	
Joint pain	6 (9.7)	2 (8.3)	4 (12.9)	2 (7.4)	
Bowel urgency	4 (12.9)	3 (12.5)	5 (16.1)	3 (11.1)	
Abdominal pain	8 (25.8)	2 (8.3)	2 (6.5)	1 (3.7)	
Embarrassment	2 (6.5)	0 (0.0)	1 (3.2)	3 (11.1)	
Others	1 (3.2)	1 (4.2)	0 (0.0)	0 (0.0)	
**Quality of life ^2^**					
SIBDQ, mean (SD)	48.9 (7.9)	51.5 (8.1)	52.5 (9.5)	54.1 (8.9)	−1.1 (−4.4, 2.2)
FR-QoL-29, mean (SD)	86.8 (23.6)	95.7 (23.9)	95.7 (29.1)	104.1 (27.9)	−2.6 (−10.3, 5.1)

Abbreviations: FR-QoL-29 = food related-quality of life, LQ = lower quartile, MET-mins/week = metabolic equivalent minutes per week, *n* = number, SIBDQ = Short inflammatory bowel disease questionnaire, UQ = upper quartile. Regression analysis only includes participants with complete data: Intervention group (*n* = 24), Control group (*n* = 27). ^1^ Separate regression models were used for each outcome, with baseline values included as a covariate and adjustments made for age, sex, season, and disease activity. ^2^ Separate regression models were used for each outcome, with baseline values included as a covariate and adjustments made for age, sex, and disease activity. SIBDQ was additionally adjusted for perceived barriers to physical activity. SIBDQ has a score range of 7–70, and the FR-QoL-29 ranges from 29 to 145, with higher scores indicating better quality of life. CI = confidence interval; SD = standard deviation.

## Data Availability

The data underlying this article will be shared on reasonable request to the corresponding author.

## Supplementary Materials

The following supporting information can be downloaded at: https://www.mdpi.com/article/10.3390/nu18050785/s1, Table S1: Baseline demographics and health characteristics of completers and dropouts.

## Abbreviations

The following abbreviations are used in this manuscript:

IBD	Inflammatory bowel disease
MD	Mean difference
SD	Standard deviation
CI	Confidence intervals
LQ	Lower quartile
UQ	Upper quartile
DXA	Dual-energy X-ray Absorptiometry
CRP	C-reactive protein
BIA	Bioimpedance analysis
CD	Crohn’s disease
UC	Ulcerative colitis
IPAQ-SF	International physical activity questionnaire - short form
MET	Metabolic equivalent task
BMI	Body mass index
CVD	Cardiovascular disease
FR-QoL	Food related-quality of life
