# Supplementary material for: A Randomised Controlled Trial Comparing the Effects of Personalised Diet and Physical Activity Intervention Versus Usual Care on Cardiometabolic Risk Factors in Adults with Inactive Inflammatory Bowel Disease
Source: Nutrients. 2026 Feb 27;18(5):785. doi: 10.3390/nu18050785 (PMC12986630; doi:10.3390/nu18050785)
Supplement: Supplementary file 1 [file nutrients-18-00785-s001.zip › nutrients-4167343-supplementary.pdf]

Supplementary Table S1. Baseline demographics and health characteristics of completers and dropouts.

	Intervention		Control	
	Completers (n = 24)	Dropouts * (n = 7)	Completers (n = 27)	Dropouts * (n = 4)
**Age, median (LQ, UQ)**	47 (43, 54)	46 (32, 47)	51 (36, 56)	37 (28, 49)
**Biological sex—Female, n (%)**	16 (66.7)	6 (85.7)	14 (51.9)	1 (25.0)
**Ethnicity, n (%)** [1]				
NZ European	22 (91.7)	6 (85.7)	24 (88.9)	4 (100.0)
Māori	1 (4.2)	0 (0.0)	2 (7.4)	1 (25.0)
Others	2 (8.3)	1 (14.3)	3 (11.1)	0 (0)
**Smoking status, n (%)**				
Active	0 (0)	1 (14.3)	1 (3.7)	0 (0)
Ex-smoker	4 (16.7)	2 (28.6)	4 (14.8)	1 (25.0)
**Vaping status- Yes, n (%)** [2]	1 (4.2)	1 (14.3)	1 (3.7)	1 (25.0)
**Comorbidities—Yes, n (%)**	10 (41.7)	4 (42.9)	14 (51.9)	1 (25.0)
High blood pressure	4 (16.7)	0 (0)	5 (18.5)	0 (0)
Diabetes	1 (4.2)	0 (0)	1 (3.7)	0 (0)
High cholesterol	1 (4.2)	0 (0)	1 (3.7)	0 (0)
Asthma	3 (12.5)	1 (14.3)	3 (11.1)	0 (0)
Arthritis	1 (4.2)	1 (14.3)	1 (3.7)	0 (0)
Others	4 (16.7)	3 (42.9)	4 (14.8)	1 (25.0)
**IBD measures**				
Disease duration, median (LQ, UQ)	11 (7, 18)	15 (10, 22)	12 (7, 20)	11 (6,15)
**Crohn's disease, n (%)**	11 (45.8)	5 (71.4)	18 (66.7)	3 (75.0)
Ileal	1 (9.1)	2 (40.0)	3 (16.7)	1 (33.3)
Colonic	2 (18.2)	1 (20.0)	6 (33.3)	0 (0)
Ileocolonic	8 (72.7)	2 (40.0)	9 (50.0)	2 (66.6)
Upper GI	0 (0)	0 (0)	0 (0)	0 (0)
**Ulcerative colitis, n (%)**	13 (54.2)	2 (28.6)	9 (33.3)	1 (25.0)
Proctitis	2 (15.4)	0 (0)	1 (11.1)	0 (0)
Left sided	5 (38.5)	1 (50.0)	5 (55.6)	1 (100.0)
Extensive	6 (46.1)	1(50.0)	3 (33.3)	0 (0)
**IBD resections—Yes, n (%)**	6 (25.0)	2 (28.6)	3 (11.1)	1 (25.0)
**Maintenance therapy, n (%)**				
None	5 (20.8)	1 (14.3)	5 (18.5)	0 (0)
Aminosalicylates	8 (33.3)	2 (28.6)	7 (25.9)	0 (0)
Immunomodulators	8 (33.3)	1 (14.3)	10 (37.0)	4 (100.0)
Biologics	9 (37.5)	4 (57.1)	13 (48.2)	1 (25.0)
**Disease activity, n (%)** [3]				
Remission	7 (29.2)	1 (14.3)	16 (59.3)	3 (75.0)
Mild	14 (58.3)	2 (28.6)	8 (29.6)	1 (25.0)
Active	3 (12.5)	4 (57.1)	3 (11.1)	0 (0)
**Inflammatory biomarkers, n (%)**				
**Faecal calprotectin** [4]				
Remission, < 150 μg/g	19 (79.2)	3 (75.0)	24 (88.9)	3 (100.0)
Mild, 150–250 μg/g	2 (0)	0 (0)	1 (3.7)	0 (0)
Active, >250 μg/g	3 (12.5)	1 (25.0)	2 (7.4)	0 (0)
**CRP**				

<5 g/L	17 (70.8)	5 (71.4)	21 (77.8)	4 (100.0)
>5 g/L	7 (29.2)	2 (28.6)	6 (22.2)	0 (0)

[1] Self-identified ethnicity was classified into three ethnic groups using the 2006 NZ census ethnicity questions and the Ministry of Health classification system. Participants could select multiple ethnicities, so column totals do not necessarily total 100%. Other ethnicities included British, European, Dutch (intervention group) and German, Scottish, European (control group). [2] Vape with nicotine content. [3] Disease activity was measured using the Harvey Bradshaw Index (HBI) for CD and the Simple Clinical Colitis Activity Index (SCCAI) for UC and IBD-unspecified. Disease activity was classified according to the following HBI scores: <5 remission, 5–7 mildly active, and ≥8 active 186; and SCCAI scores: ≤2 remission, <5 mildly active, and ≥5 active. [4] Missing data from dropouts: intervention (n = 3) and control group (n = 1). * Does not include participants excluded: intervention (n = 1), control (n = 1). LQ= lower quartile; UQ= upper quartile; n=number; BMI= body mass index; NZ= new zealand; IBD= inflammatory bowel disease; GI= gastrointestine; CRP= C-reactive protein.
